# EASIX as a moderately effective prognostic marker for mortality in severe acute pancreatitis: a retrospective study

**DOI:** 10.3389/fmed.2026.1854703

**Published:** 2026-07-07

**Authors:** Lvyuan Shi, Lili Cao, Dingyuan Wan, Lietao Wang, Ping Li, Siyuan Chen, Zhongwei Zhang, Min He

**Affiliations:** 1 Department of Critical Care Medicine, West China Hospital, Sichuan University, Chengdu, Sichuan, China; 2 Institute of Basic Medical College, Chengdu University, Chengdu, China

**Keywords:** EASIX, ICU, mortality, severe acute pancreatitis, short-term prognosis

## Abstract

**Introduction:**

The endothelial activation and stress index (EASIX) has been proposed as a surrogate laboratory index reflecting endothelial activation and stress. This study aimed to evaluate the prognostic value of EASIX in patients with severe acute pancreatitis (SAP).

**Methods:**

This retrospective observational study analyzed 340 SAP patients hospitalized between December 2015 and December 2023. EASIX was calculated as LDH (U/L) × creatinine (mg/dL) / platelet count (10^9^/L). Regression analyses identified mortality predictors, which were incorporated into a prognostic model. Receiver operating characteristic (ROC) analysis compared the predictive performance of EASIX and the developed model.

**Results:**

The overall mortality rate was 28.82%. Non-survivors exhibited significantly elevated EASIX levels compared to survivors (*p* < 0.001). Multivariate analysis identified EASIX as an independent predictor of mortality (*p* = 0.004), along with age, APACHE II score, white blood cell count, and shock. The predictive model incorporating these factors achieved an AUC of 0.77 (sensitivity 0.73, specificity 0.70), outperforming EASIX alone (AUC = 0.70).

**Conclusion:**

EASIX is a moderately effective prognostic marker in SAP, with performance comparable to APACHE II. The developed predictive model incorporating EASIX shows improved accuracy over EASIX alone for mortality risk stratification, but external validation is needed before clinical application.

## Introduction

Acute pancreatitis is a prevalent form of clinical acute abdomen, characterized by complexity and challenges in early-stage prediction. Approximately 80% of patients present with mild to moderate disease, with no organ failure exceeding 48 h. Nevertheless, one-fifth of patients progress to severe disease (1), which can be life-threatening (2). The revised Atlanta classification divides acute pancreatitis severity into mild, moderately severe, and severe categories (3). Mild acute pancreatitis lacks both organ failure and local or systemic complications. Moderately severe acute pancreatitis is characterized by transient organ failure (resolving within 48 h) and/or the presence of local or systemic complications. Severe acute pancreatitis is strictly defined by persistent organ failure lasting longer than 48 h. The hospital mortality rate for severe acute pancreatitis has reached 15% (4), increasing further if it is complicated by infection (5). Multiple organ failure (MOF) is recognized as a critical complication of severe acute pancreatitis, with at least half of fatalities attributed to MOF (6). Systemic endothelial dysfunction has emerged as the primary contributor to MOF in severe acute pancreatitis patients. This dysfunction involves endothelial cell activation, increased adhesion molecule production, increased endothelial barrier permeability, and intravascular coagulation, collectively underscoring endothelial dysfunction as a pivotal pathophysiological feature of severe acute pancreatitis (SAP) (7–9).

The Endothelial Activation and Stress Index (EASIX), formulated as LDH (U/L) × creatinine (mg/dL) / platelet count (10^9^/L), represents a laboratory-based prognostic indicator (10). Originally devised to predict mortality in acute graft-versus-host disease (GVHD) patients (11), EASIX has subsequently been employed to explore prognostic factors across various conditions, including myelodysplastic syndrome, diffuse large B-cell lymphoma, small-cell lung cancer, demonstrating its efficacy as a predictive index (12–14). A study by Felix Korell revealed EASIX as a prognostic marker for assessing the risk of sepsis following allogeneic stem cell transplantation (allo SCT), underscoring its relevance in life-threatening emergencies associated with endothelial cell disorders (15). Moreover, a study conducted by Xu confirmed a significant association between elevated EASIX and an increased risk of 28- and 90-day all-cause mortality in sepsis patients (16). Although EASIX has been proposed as a surrogate marker of endothelial stress, direct mechanistic evidence linking EASIX to endothelial dysfunction in SAP is lacking. Nonetheless, its association with outcomes in conditions characterized by endothelial injury (e.g., GVHD, sepsis) suggests potential relevance of promptly identifying biomarkers for SAP patients with an unfavorable prognosis.

A recent retrospective study using the MIMIC-IV database reported that EASIX was associated with mortality in ICU patients with acute pancreatitis, with an AUC of 0.68 (17). However, that study included all severity categories of acute pancreatitis (mild, moderate, severe) and did not develop a multivariable prediction model or provide a bedside clinical tool. Therefore, to extend these findings, we focused specifically on severe acute pancreatitis (SAP) according to the Revised Atlanta Classification and aimed to (1) validate the prognostic value of EASIX in a well-defined SAP cohort, (2) construct and internally validate a multivariable predictive model incorporating EASIX and other independent predictors, and (3) provide a practical nomogram for bedside risk stratification.

## Methods

### Study design

This retrospective study included all patients who were diagnosed with severe acute pancreatitis at the Department of Critical Care Medicine, West China Hospital of Sichuan University (Chengdu, China) from December 2015 to December 2023. The diagnosis of SAP was established based on the Revised Atlanta Classification (3), requiring the presence of both: (1) characteristic abdominal pain with serum lipase or amylase levels at least three times the upper limit of normal; and (2) persistent organ failure (lasting >48 h) involving the respiratory, cardiovascular, or renal systems. We initially screened all adult patients (≥18 years) with a primary diagnosis of SAP upon ICU admission.

Etiology of acute pancreatitis was determined by standard clinical criteria: hypertriglyceridemia (serum triglycerides > 11.3 mmol/L or >5.6–11.3 mmol/L with lactescent serum), alcoholic (daily intake >50 g for men or >40 g for women), biliary (cholelithiasis or sludge on abdominal ultrasound), idiopathic (no identifiable cause after routine laboratory and imaging workup), and other (including drug-induced, post-ERCP, traumatic, autoimmune, and rare causes). Organ failure was defined and assessed according to the modified Marshall scoring system as recommended by the Revised Atlanta Classification. This system evaluates three organ systems: Respiratory failure: PaO2/FiO2 ratio < 300. Renal failure: Serum creatinine ≥ 1.9 mg/dL (170 μmol/L) or a doubling from baseline. Cardiovascular failure: Systolic blood pressure < 90 mmHg not responsive to fluid resuscitation, or need for vasopressor support. Inclusion criteria were: (1) age ≥18 years, (2) diagnosis of severe acute pancreatitis according to the Revised Atlanta Classification (3), and (3) availability of complete laboratory data for EASIX calculation within 24 h of ICU admission. No patients were excluded based on consciousness level, cooperation, or resuscitation status. After applying these criteria, a total of 340 patients diagnosed with severe acute pancreatitis were ultimately included in the study (Supplementary Figure S1).

This retrospective study was conducted in accordance with the Declaration of Helsinki and was approved by the Ethics Committee of West China Hospital, Sichuan University (Approval NO: 2021–1,694). Given the retrospective nature of the study using anonymized clinical data, the requirement for written informed consent was waived by the ethics committee.

### Study variables

Data pertaining to baseline characteristics, laboratory test results, and outcomes were retrieved from the West China Hospital of Sichuan University database. Laboratory variables were derived from the initial examination results of severe acute pancreatitis (SAP) patients upon their admission to the Intensive Care Unit (ICU). These variables included hemoglobin level, white blood cell count, platelet count, lactate dehydrogenase level, prothrombin time, procalcitonin level and serum creatinine level. EASIX scores were computed using the formula: lactate dehydrogenase (U/L) × creatinine (mg/dL) / platelet count (10^9/L), based on the first laboratory measurements obtained after ICU admission. Disease severity was assessed using the Acute Physiology and Chronic Health Evaluation II (APACHE II) score, which was automatically calculated and populated by the electronic medical record system using data captured from routine clinical workflows within the first 24 h of ICU admission. The primary outcome was ICU mortality, defined as all-cause death during the index ICU admission. All patients remained in the same hospital until ICU discharge or death; no inter-hospital transfers occurred during the ICU stay. Therefore, outcome status was known for all patients. The incidence of shock, respiratory failure, renal failure, duration of Mechanical Ventilation, length of ICU stay, and length of hospital stay were compared between survivors and non-survivors.

### Statistical analysis

Missing data were handled using multiple imputation by chained equations (MICE) under the assumption of missing at random, generating five imputed datasets for analysis. Missingness was minimal for all variables included in the final model (<5% for most, none >15%). A complete-case sensitivity analysis yielded results consistent with the imputed model, confirming that imputation did not materially affect the conclusions. The normality of continuous variables was assessed using the Kolmogorov–Smirnov test. Data are presented as medians with interquartile ranges for non-normally distributed variables and as means ± standard deviations for normally distributed variables. Between-group comparisons (survivors vs. non-survivors) were performed using the Mann–Whitney U test for non-parametric data and Student’s t-test for parametric data. Categorical variables were compared using the Chi-square test or Fisher’s exact test, as appropriate. The restricted cubic spline (RCS) method was used to generally explore the relationship between EASIX and mortality of SAP. Variables that showed a significant association with mortality in the univariate logistic regression analysis (using a threshold of *p* < 0.05 for inclusion to avoid excluding potentially important confounders) were entered into a multivariate logistic regression model. All five variables were entered simultaneously without any stepwise selection. A prognostic nomogram was developed based on the final multivariate model to facilitate clinical application. Model performance was evaluated using receiver operating characteristic (ROC) curve analysis, which compared the predictive accuracy of EASIX alone versus the full prognostic model. To assess potential multicollinearity among the independent variables in the final multivariable logistic regression model, variance inflation factor (VIF) was calculated using the ‘car’ package in R. A VIF value greater than 5 was considered indicative of significant collinearity. To evaluate the statistical differences in the discriminatory ability among different predictive indicators, the DeLong test was used. Calibration was assessed using a calibration plot. A two-sided *p*-value < 0.05 was considered statistically significant. All analyses were conducted using SPSS (version 25.0) and R (version 4.2.2).

## Results

A total of 340 patients diagnosed with severe acute pancreatitis and initially admitted to the Intensive Care Unit (ICU) between December 2015 and December 2023. Table 1 outlined the baseline characteristics of the study cohorts. The study included 340 patients, of whom 242 (71.18%) survived and 98 (28.82%) died. Significant differences were observed between survivors and non-survivors in the following variables: age, heart rate, systolic blood pressure, diastolic blood pressure, APACHE II score, total length of stay, white blood cell count, platelet count, lactate dehydrogenase, prothrombin time, procalcitonin, serum creatinine, duration of mechanical ventilation, shock, respiratory failure, and renal failure (all *p* < 0.05). In contrast, no significant differences were found in body temperature, ICU length of stay, hemoglobin levels, male gender, or etiology (all *p* > 0.05).

**Table 1:** Demographic and baseline characteristics of patients with severe acute pancreatitis.

Variables	Total (*n* = 340)	Survivors (*n* = 242, 71.18%)	Non-survivors (*n* = 98, 28.82%)	*p*
Age (year)	47.00 (36.75, 54.00)	45.00 (34.00, 54.00)	50.00 (42.00, 58.75)	**0.002**
Gender, male, *n* (%)	226 (66.47)	159 (65.70)	67 (68.37)	0.637
Etiology, *n* (%)				0.161
Hypertriglyceridemia, *n* (%)	55 (16.18)	46 (19.01)	9 (9.18)	
Alcoholic, *n* (%)	51 (15.00)	36 (14.88)	15 (15.31)	
Biliary, *n* (%)	20 (5.88)	16 (6.61)	4 (4.08)	
Idiopathic, *n* (%)	80 (23.53)	51 (21.07)	29 (29.59)	
Other, n (%)	134(39.41)	93 (38.43)	41 (41.84)	
Admission vital signs				
Body temperature (°C)	37.00 (36.50, 37.60)	37.00 (36.50, 37.60)	37.00 (36.50, 37.50)	0.750
Heart rate (s^−1^)	117.00 (99.00, 133.25)	114.00 (95.00, 131.75)	126.00 (109.25, 138.00)	**0.002**
Systolic blood pressure (mmHg)	124.00 (106.00, 140.00)	125.00 (110.00, 140.75)	120.50 (100.25, 135.00)	**0.015**
Diastolic blood pressure (mmHg)	72.00 (61.75, 84.00)	74.00 (64.00, 85.00)	70.00 (60.00, 79.75)	**0.022**
Apache II	19.66 (14.00, 25.00)	18.00 (12.25, 23.00)	22.00 (19.00, 29.00)	**<0.001**
Admission laboratory data				
Hemoglobin (g/L)	109.00 (85.00, 143.25)	112.00 (88.00, 142.00)	98.50 (82.25, 146.50)	0.147
White blood cell (10^9^/L)	12.55 (8.91, 16.74)	11.86 (8.55, 16.40)	13.31 (10.49, 18.25)	**0.030**
Platelet (10^9^/L)	164.50 (107.75, 235.25)	177.50 (121.00, 245.75)	131.50 (80.00, 202.75)	**<0.001**
Lactate dehydrogenase (U/L)	498.00 (274.25, 758.50)	450.50 (235.50, 696.39)	622.50 (366.50, 1063.50)	**<0.001**
Prothrombin time (s)	13.80 (12.80, 15.40)	13.60 (12.70, 15.20)	14.40 (13.22, 16.20)	**0.006**
Procalcitonin (ng/ml)	1.92 (0.44, 8.02)	1.31 (0.32, 6.07)	3.67 (1.45, 13.74)	**<0.001**
Serum creatinine (mg/dL)	1.06 (0.66, 2.16)	0.85 (0.59, 1.83)	1.60 (0.95, 3.19)	**<0.001**
EASIX	3.15 (1.10, 12.74)	2.29 (0.82, 8.37)	6.90 (2.92, 22.63)	**<0.001**
Shock, *n* (%)	193 (56.76)	117 (48.35)	76 (77.55)	**<0.001**
Respiratory failure, *n* (%)	205 (60.29)	122 (50.41)	83 (84.69)	**<0.001**
Renal failure, *n* (%)	104 (30.59)	49 (20.25)	55 (56.12)	**<0.001**
Duration of mechanical ventilation (hours)	166.50 (0.00, 288.00)	96.00 (0.00, 215.28)	213.13 (71.00, 408.00)	**<0.001**
ICU length of stay (days)	14.00 (7.00, 24.00)	13.00 (7.00, 24.75)	14.50 (7.00, 24.00)	0.626
Total length of stay (days)	22.00 (14.00, 34.00)	24.00 (16.00, 39.00)	17.00 (9.00, 25.75)	**<0.001**

Bold values indicate statistical significance (*p* < 0.05).

**Figure 1:**
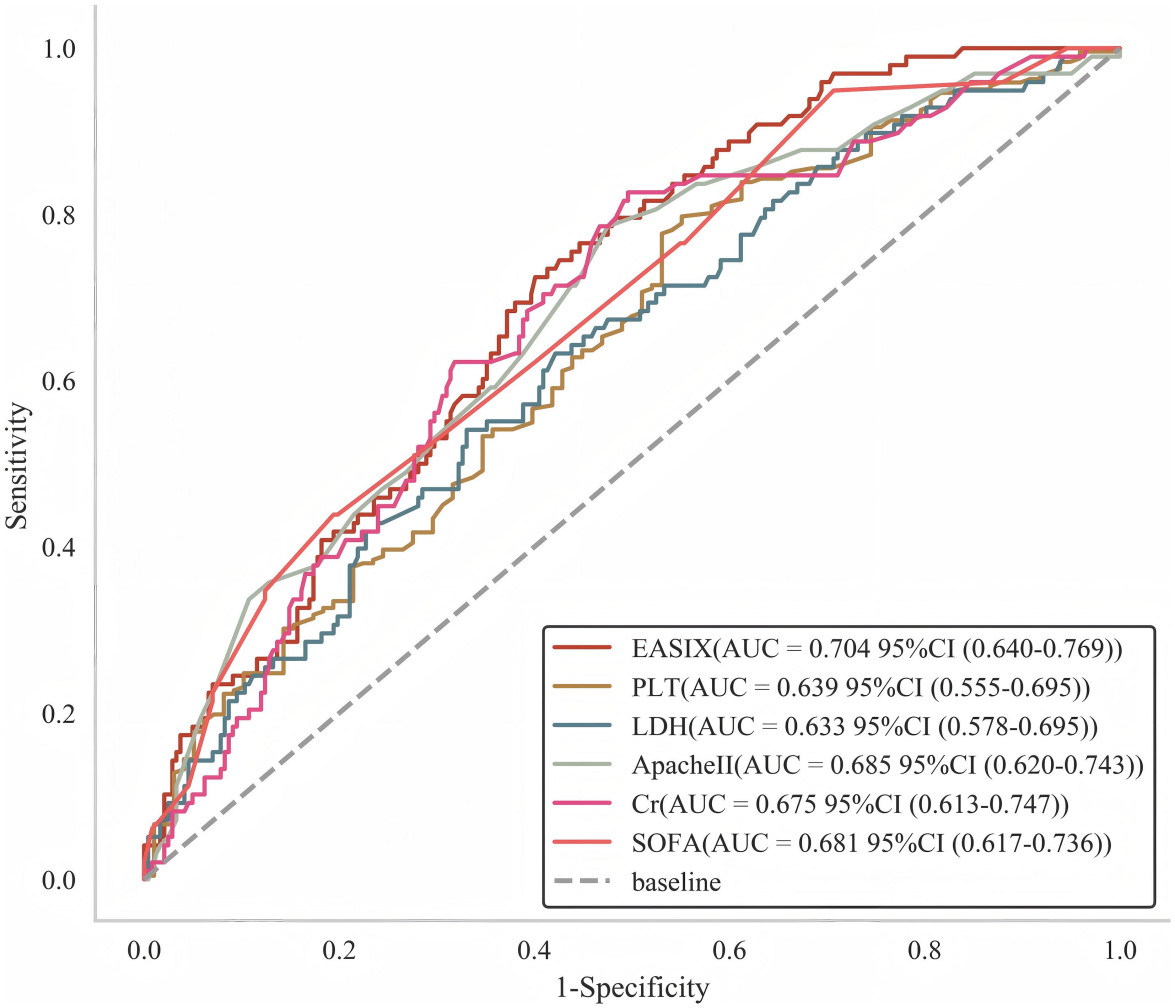
Relationship between the EASIX and mortality of SAP patients. The curve illustrates the odds ratio (OR) for mortality as a function of the Endothelial Activation and Stress Index (EASIX) value. The shaded area represents the 95% confidence interval. The overall association was significant (P for overall < 0.001), indicating that the risk increases progressively with higher EASIX levels.

The RCS showed the EASIX was positively related with the mortality of SAP (Figure 1). Of the variables analyzed by univariate logistic regression, ten were significantly associated with mortality (*p* < 0.05). These included Age (OR 1.03, 95% CI 1.01–1.05, *p* = 0.001), APACHE II score (OR 1.09, 95% CI 1.05–1.13, *p* < 0.001), White blood cell count (OR 1.04, 95% CI 1.01–1.08, *p* = 0.021), Prothrombin time (OR 1.09, 95% CI 1.02–1.16, *p* = 0.012), Procalcitonin (OR 1.02, 95% CI 1.01–1.03, *p* = 0.006), EASIX (OR 1.01, 95% CI 1.01–1.02, *p* = 0.001), Shock (OR 3.69, 95% CI 2.16–6.32, *p* < 0.001), and Duration of Mechanical Ventilation (OR 1.01, 95% CI 1.01–1.01, *p* = 0.007). Systolic and Diastolic blood pressure also showed significant but minimal protective effects (OR 0.99 and 0.98, respectively). Multivariable logistic regression with the five pre-specified predictors identified all five as independent predictors of mortality (Table 2). These were: Age (adjusted OR 1.03, 95% CI 1.01–1.05, *p* = 0.003), APACHE II score (adjusted OR 1.05, 95% CI 1.01–1.09, *p* = 0.009), White blood cell count (adjusted OR 1.04, 95% CI 1.01–1.08, *p* = 0.041), EASIX (adjusted OR 1.01, 95% CI 1.01–1.02, *p* = 0.004), and Shock (adjusted OR 2.98, 95% CI 1.66–5.35, *p* < 0.001). Because EASIX is right-skewed, we also calculated the odds ratio per interquartile range (IQR) increase (from the 25th to 75th percentile), which was 1.12 (95% CI 1.05–1.20). Notably, the strength of association for Shock and the APACHE II score was attenuated in the multivariate model compared to the univariate analysis (Table 2).

**Table 2:** Univariate and multivariate logistic regression analysis for exploring risk factors of mortality in SAP patients.

Variables	Univariate logistic regression			Multivariate logistic regression		
	OR	95% CI	*p*	OR	95% CI	*p*
Age	1.03	1.01 ~ 1.05	**0.001**	1.03	1.01 ~ 1.05	**0.003**
Gender, male	1.13	0.68 ~ 1.86	0.637			
Body temperature	1.07	0.86 ~ 1.33	0.523			
Heart rate	1.00	1.00 ~ 1.00	0.538			
Systolic blood pressure	0.99	0.98 ~ 1.00	**0.022**	1.00	0.98 ~ 1.01	0.669
Diastolic blood pressure	0.98	0.97 ~ 1.00	**0.016**	0.99	0.97 ~ 1.02	0.610
Apache II	1.09	1.05 ~ 1.13	**<0.001**	1.05	1.01 ~ 1.09	**0.009**
Hemoglobin	1.00	0.99 ~ 1.00	0.313			
White blood cell	1.04	1.01 ~ 1.08	**0.021**	1.04	1.00 ~ 1.08	**0.042**
Prothrombin time	1.09	1.02 ~ 1.16	**0.012**	1.05	1.00 ~ 1.10	0.067
Procalcitonin	1.02	1.01 ~ 1.03	**0.006**	1.01	0.99 ~ 1.02	0.534
EASIX	1.01	1.01 ~ 1.02	**0.001**	1.01	1.00 ~ 1.02	**0.009**
Shock	3.69	2.16 ~ 6.32	**<0.001**	2.48	1.35 ~ 4.54	**0.003**
Duration of mechanical ventilation	1.00	1.00 ~ 1.00	**0.007**	1.00	1.00 ~ 1.00	0.166

Bold values indicate statistical significance (*p* < 0.05).

**Figure 2:**
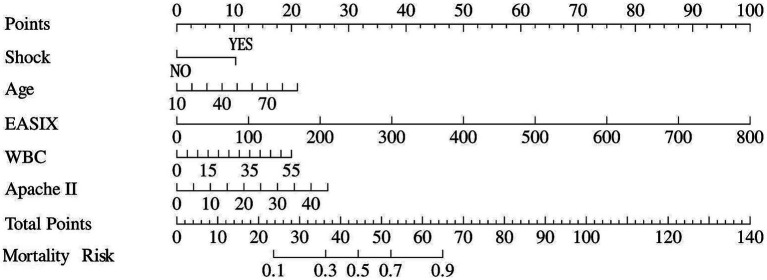
Comparative ROC analysis of EASIX, its components, and APACHE II for mortality prediction in severe acute pancreatitis. The area under the curve (AUC) for each predictor is shown in parentheses. EASIX demonstrated an AUC of 0.704 (95% CI: 0.640–0.769), which was comparable to APACHE II (AUC 0.685) and superior to its individual components.

The predictive performance of individual biomarkers and clinical scores for mortality in severe acute pancreatitis (SAP) patients is summarized in Table 3 and illustrated in Figure 2. The areas under the receiver operating characteristic curve (AUC) for lactate dehydrogenase (LDH), platelet count (PLT), serum creatinine (Cr), the Endothelial Activation and Stress Index (EASIX), SOFA score, and the APACHE II score were 0.633 (95% CI: 0.578–0.695), 0.639 (95% CI: 0.555–0.695), 0.675 (95% CI: 0.613–0.747), 0.704 (95% CI: 0.640–0.769), 0.681(95% CI: 0.617–0.736), and 0.685 (95% CI: 0.620–0.743), respectively. EASIX demonstrated a numerically higher AUC (0.70) than APACHE II (0.68) and SOFA (0.68), but the differences were not statistically significant (DeLong *p* = 0.618 and *p* = 0.489, respectively). To assess the statistical differences in discriminatory ability among various predictive indicators, we performed pairwise comparisons of the area under the receiver operating characteristic curve (AUC) using the DeLong test. The results showed no significant differences in predictive performance between EASIX and the APACHE II score (*p* = 0.618) or the SOFA score (p = 0.489).

**Table 3:** Predictive performance of EASIX and the predictive model for mortality in SAP patients.

Variables	AUC	95% CI	Sensitivity	Specificity	Youden index	Cut-off value
LDH	0.63	0.57–0.70	0.58	0.63	0.21	513.5
PLT	0.64	0.57–0.70	0.20	0.55	−0.25	112.5
Cr	0.67	0.61–0.74	0.50	0.83	0.33	0.855
EASIX	0.70	0.65–0.76	0.60	0.72	0.32	3.275
APACHE II	0.68	0.62–0.75	0.52	0.79	0.31	18.5
Predictive model	0.77	0.72–0.83	0.73	0.70	0.43	0.301

**Figure 3:**
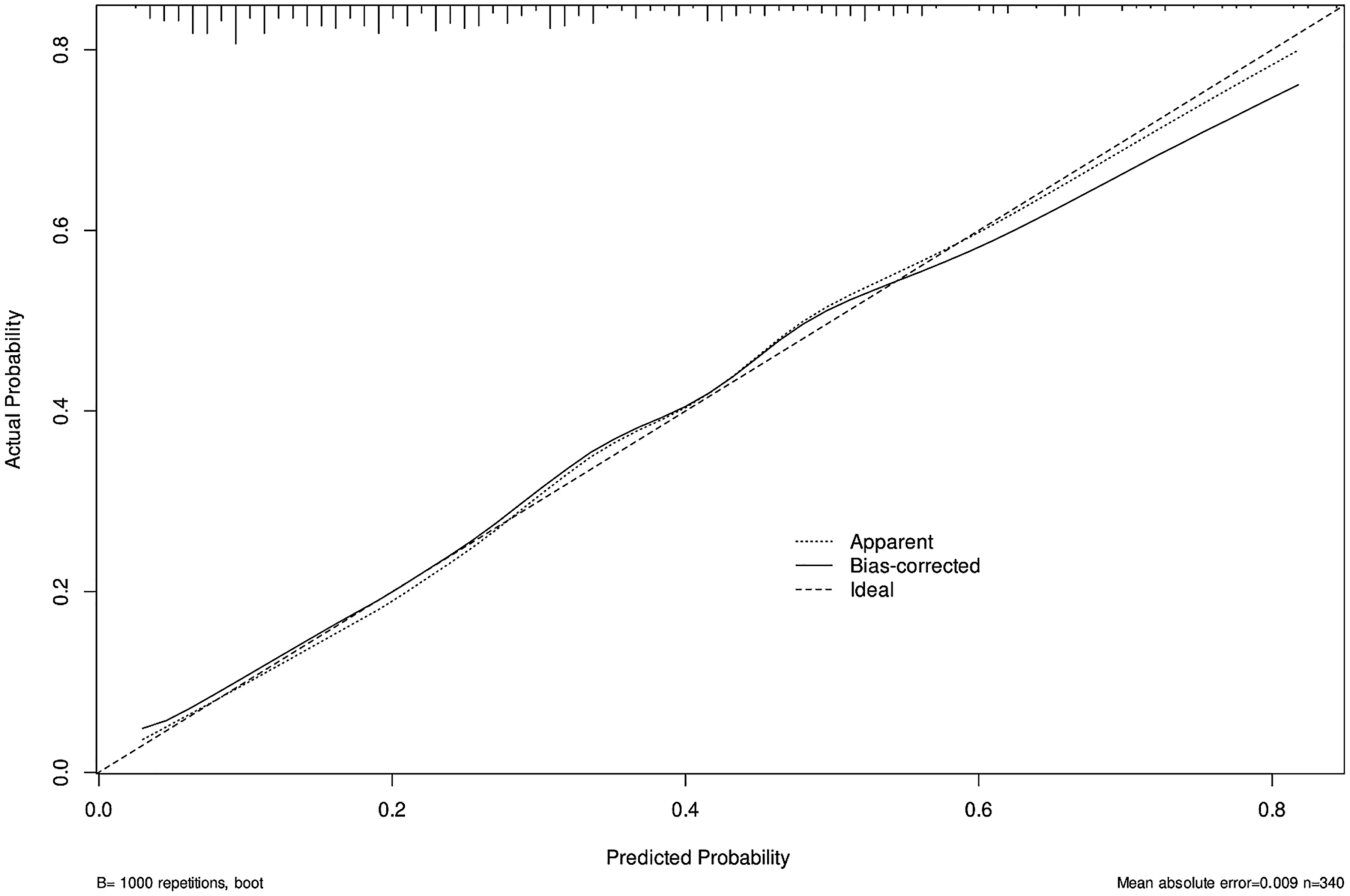
The nomogram of the predictive model for mortality in SAP patients. The nomogram integrates five independent predictors: shock (yes/no), age (years), EASIX, white blood cell count (WBC, ×10^9^/L), and APACHE II score. To use the nomogram, locate the patient’s value for each predictor on the corresponding axis, draw a vertical line to the “Points” scale to obtain the points for each variable, sum these points to obtain the total points, and then project the total points onto the “Mortality Risk” scale to estimate the individual probability of ICU mortality.

**Figure 4:**
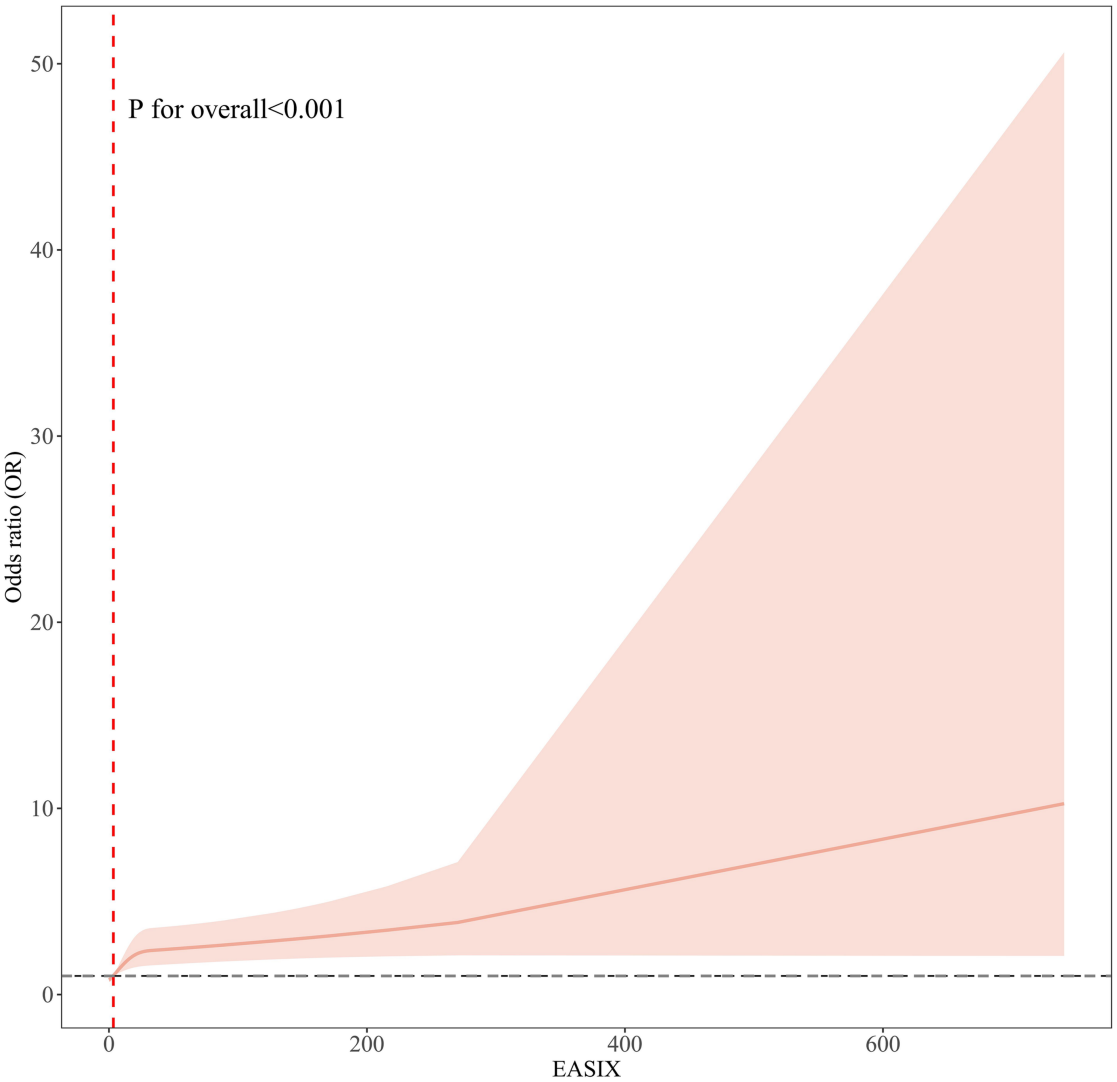
The calibration plot of the predictive model for mortality in SAP patients. The plot compares the predicted probability of mortality from the nomogram (*x*-axis) with the observed probability (*y*-axis). The diagonal dashed line represents perfect calibration (ideal). The solid line shows the apparent calibration, and the bias-corrected line (obtained from bootstrapping) indicates the model’s performance after internal validation. The close agreement between the lines suggests good calibration of the model.

Finally, a comprehensive predictive model was developed by incorporating the independent risk factors identified through multivariate analysis, namely Shock, Age, EASIX, White Blood Cell count (WBC), and the APACHE II score. Multicollinearity diagnostics revealed no significant collinearity among the five independent predictors (all VIF < 2). This integrated model achieved a superior predictive performance, with an AUC of 0.77, a sensitivity of 0.73, and a specificity of 0.70 (Table 3). For convenient clinical application, this model was visually presented as a nomogram (Figure 3). Furthermore, the DeLong test was applied to compare the predictive performance of the integrated model (AUC 0.77) versus EASIX alone (AUC 0.70). The difference was statistically significant (*p* = 0.041), indicating that the model provides a superior discriminatory ability for mortality over the single EASIX index. We also evaluated a reduced model excluding APACHE II (using age, WBC, EASIX, and shock only), which achieved an AUC of 0.74 (95% CI: 0.69–0.79), still significantly higher than EASIX alone. The calibration curve (Figure 4) further indicated a strong agreement between the predicted probabilities of mortality by the model and the actual observed outcomes. Decision-curve analysis (Supplementary Figure S2) confirmed that the predictive model provided a positive net benefit across clinically relevant threshold probabilities.

## Discussion

Acute severe pancreatitis is a formidable condition initiated by the activation of trypsinogen, culminating in inflammatory responses characterized by pancreatic self-digestion, edema, bleeding, and potential necrosis (18–20). Its gravest manifestations encompass hypotension, organ insufficiency, adult respiratory distress syndrome (ARDS), multiple organ failure (MOF), and ultimately, fatality (21, 22). The hospital mortality rate for patients grappling with severe acute pancreatitis reaches a staggering 15% (4). Systemic complications stand out as predominant features of SAP and are universally acknowledged as critical determinants of prognosis (23–25). Approximately 20% of SAP patients experience organ failure within 72 h of onset, with some presenting organ failure or multiple organ failure upon admission (26).

The Endothelial Activation and Stress Index (EASIX), established as a laboratory-based prognostic indicator, is defined by the formula: lactate dehydrogenase (mg/dL) × creatinine (mg/dL) / platelet count (10^9 cells / L). Initially proven valuable in predicting mortality in acute graft-versus-host disease (GVHD) (11), EASIX has also demonstrated efficacy as a prognostic biomarker for COVID-19 mortality (27). Ongoing investigations have associated EASIX with mortality in low- and medium-risk patients diagnosed with myelodysplastic syndrome and multiple myeloma (12, 28). Furthermore, EASIX serves as an independent predictor of 28-day and 29-day all-cause mortality in sepsis patients (16). Consequently, the Endothelial Activation and Stress Index (EASIX) emerges as a reliable biomarker indicative of endothelial dysfunction. Endothelial cells, pervasive throughout the human body, play a crucial role in the pathophysiological processes of severe acute pancreatitis (SAP). Arranged in the entire circulatory system lumen, endothelial dysfunction induced by SAP impairs endothelial function and disrupts pancreatic microcirculation (29). Endothelial damage results in fluid extravasation, hypovolemia, hypotension, elevated abdominal pressure, severe renal vasoconstriction, hypercoagulable states, and glomerular fibrin deposition, ultimately leading to acute kidney injury (AKI) (30). Endothelial cell injury stands as a common cause of acute lung injury (ALI) associated with SAP (31), with Acadesine demonstrating efficacy in alleviating acute pancreatitis-related lung injury by mediating the barrier protection of pulmonary microvascular endothelial cells (32). Systemic endothelial dysfunction remains the principal catalyst for multiple organ failure in SAP patients. Therefore, prompt identification of biomarkers for SAP patients with an adverse prognosis holds paramount importance. We acknowledge that EASIX components also reflect non-specific organ injury and inflammation; thus, its prognostic value should not be equated solely with endothelial dysfunction. Nonetheless, prior validation studies support EASIX as a clinically useful integrative index of endothelial stress.

The EASIX, comprised of three key components: lactate dehydrogenase, creatinine, and platelet count, holds significance in predicting the prognosis of severe acute pancreatitis (SAP). Lactate dehydrogenase (LDH), a constituent of the EASIX, is recognized in the Ransons standard, one of the earliest scoring systems for assessing acute pancreatitis severity (33). LDH elevation is observed early in tissue injury, necrosis, hypoxia, hemolysis, or malignancy (34–36), and has been identified as a prognostic factor for severe acute pancreatitis (according to the 1992 Atlanta standard), pancreatic necrosis, infection, and overall pancreatitis-related mortality (37, 38). The collective evaluation of serum markers, including C-reactive protein (CRP), procalcitonin (PCT), interleukin-6 (IL-6), and LDH, proves valuable in determining the severity of acute pancreatitis (39), as demonstrated in a large Italian study where CR > 2.2 mg/dL within 24 h correlated with increased mortality and pancreatic necrosis (40). Peak creatinine exceeding 3 mg/dL emerges as a clinical feature and predictor of mortality in acute renal injury complicating severe acute pancreatitis (41, 42). Platelet count, the third component of EASIX, has been associated with intravascular and extravascular platelet accumulation in acute pancreatitis (43). Platelet activation is implicated in inflammatory reactions (44), suggesting a role in the pathogenesis and complications of severe acute pancreatitis, as highlighted in Osada’s study (45). The combined assessment of these three elements within EASIX appears to offer a synergistic effect in prognosticating SAP, affirming the feasibility of EASIX as a reliable biological indicator for severe acute pancreatitis prognosis.

Several clinical scores have been developed to assess severity and mortality in acute pancreatitis, including Ranson, Balthazar/CTSI, SOFA, APACHE II, and Marshall. However, each has specific limitations that may hinder routine bedside application, particularly in the early (<24 h) phase. The Ranson score requires laboratory data collected over 48 h; the Balthazar score and CT severity index depend on contrast-enhanced CT, which is not always emergently available; and the SOFA score is often calculated sequentially rather than at a single time point. The full APACHE II score traditionally requires manual collection of 12 physiological variables over 24 h, which can be time-consuming (46). The EASIX score, incorporating only three parameters—lactate dehydrogenase, creatinine, and platelet count—offers a simple yet effective means of evaluating the short-term prognosis of patients with severe acute pancreatitis (SAP). This scoring system stands out for its objectivity and feasibility, presenting a promising tool for assessing the short-term prognosis of SAP patients. EASIX demonstrated predictive performance comparable to the established APACHE II score (DeLong’s test, *p* = 0.618), while offering the practical advantage of being derived from three routinely available laboratory parameters obtained at a single time point. The streamlined nature of the EASIX score makes it a practical option for timely identification of patients with a poor prognosis, enabling the implementation of measures to standardize diagnostic and treatment approaches. This, in turn, holds the potential to reduce mortality rates and improve the overall prognosis of patients grappling with severe acute pancreatitis. Recent studies have extended the prognostic utility of EASIX to cardiovascular settings. Özlek et al. demonstrated that admission EASIX independently predicts in-hospital mortality in STEMI (AUC 0.810) (47) and in acute decompensated heart failure with reduced ejection fraction (AUC 0.751) (48). These findings, together with our results in SAP, support the broad pathophysiological significance of EASIX-reflected endothelial dysfunction across systemic inflammatory and cardiovascular disorders.

This study has several limitations. First, the single-center design and modest sample size may introduce selection bias and limit generalizability. Second, we assessed EASIX only at ICU admission; serial measurements could provide additional prognostic information but were not systematically available. Third, due to the retrospective design, several clinically relevant variables were missing or incompletely recorded: CRP, lactate, fluid balance, vasopressor dose (only binary shock was available), body mass index, comorbidities, infection status, pancreatic necrosis, and CT severity indices. Consequently, we could not adjust for these potential confounders or compare EASIX with other established prognostic scores (e.g., BISAP, Ranson, Marshall, CTSI). Fourth, our primary outcome was ICU mortality, not hospital or 28−/90-day mortality; deaths after ICU discharge were not captured. Fifth, we did not perform internal validation of discrimination (e.g., bootstrap optimism correction or cross-validation), and external validation in an independent cohort is lacking. Therefore, the reported AUC (0.77) may be optimistic, and the model is not ready for clinical use. Prospective multicenter studies with standardized data collection and external validation are needed to confirm the independent prognostic value of EASIX and its potential role in clinical practice.

## Conclusion

EASIX is a moderately effective prognostic marker in SAP, with performance comparable to APACHE II. The predictive model combining EASIX with age, APACHE II, white blood cell count, and shock shows significantly better discrimination than EASIX alone. However, external validation is required before any clinical application.

## Data Availability

The original contributions presented in the study are included in the article/Supplementary material, further inquiries can be directed to the corresponding authors.

## Supplementary material

SUPPLEMENTARY FIGURE S1 Patient flow diagram.

SUPPLEMENTARY FIGURE S2 Decision curve analysis of the predictive model for ICU mortality in patients with severe acute pancreatitis.

## Glossary

EASIX: Endothelial Activation and Stress Index
SAP: Severe Acute Pancreatitis
AP: Acute Pancreatitis
MOF: Multiple Organ Failure
GVHD: Graft-Versus-Host Disease
ICU: Intensive Care Unit
RCS: Restricted Cubic Spline
ROC: Receiver Operating Characteristic
AUC: Area Under the Curve
APACHE II: Acute Physiology and Chronic Health Evaluation II
WBC: White Blood Cell Count
LDH: Lactate Dehydrogenase
PLT: Platelet Count
Cr: Serum Creatinine
AKI: Acute Kidney Injury
ALI: Acute Lung Injury
ARDS: Adult Respiratory Distress Syndrome
CRP: C-Reactive Protein
PCT: Procalcitonin
IL-6: Interleukin-6
SOFA: Sequential Organ Failure Assessment
