# Supplementary figures and images for: EASIX as a moderately effective prognostic marker for mortality in severe acute pancreatitis: a retrospective study
Source: Front Med (Lausanne). 2026 Jul 7;13:1854703. doi: 10.3389/fmed.2026.1854703 (PMC13386221; doi:10.3389/fmed.2026.1854703)

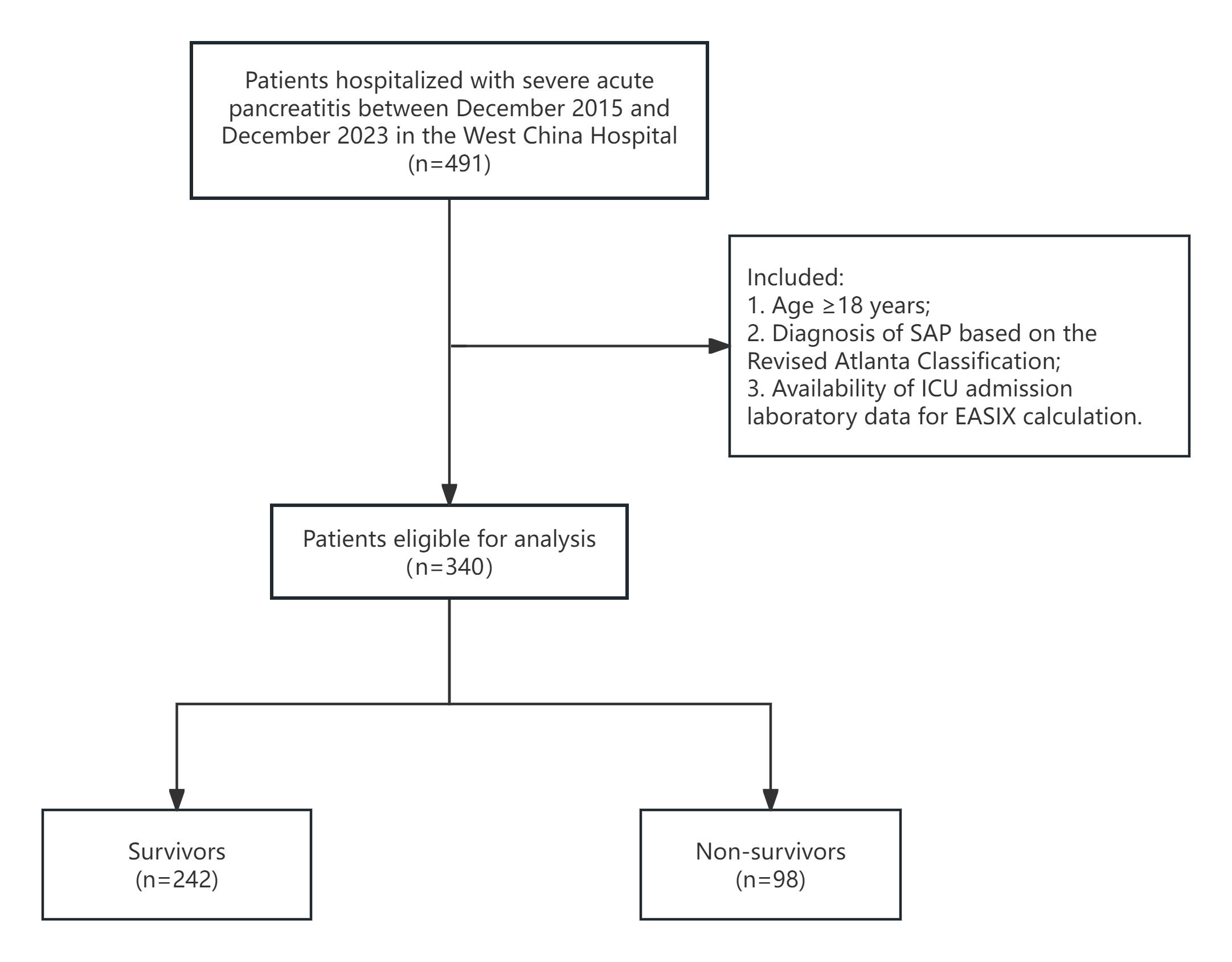

Supplement: SUPPLEMENTARY FIGURE S1 — Patient flow diagram. [file Figure_1.JPEG]

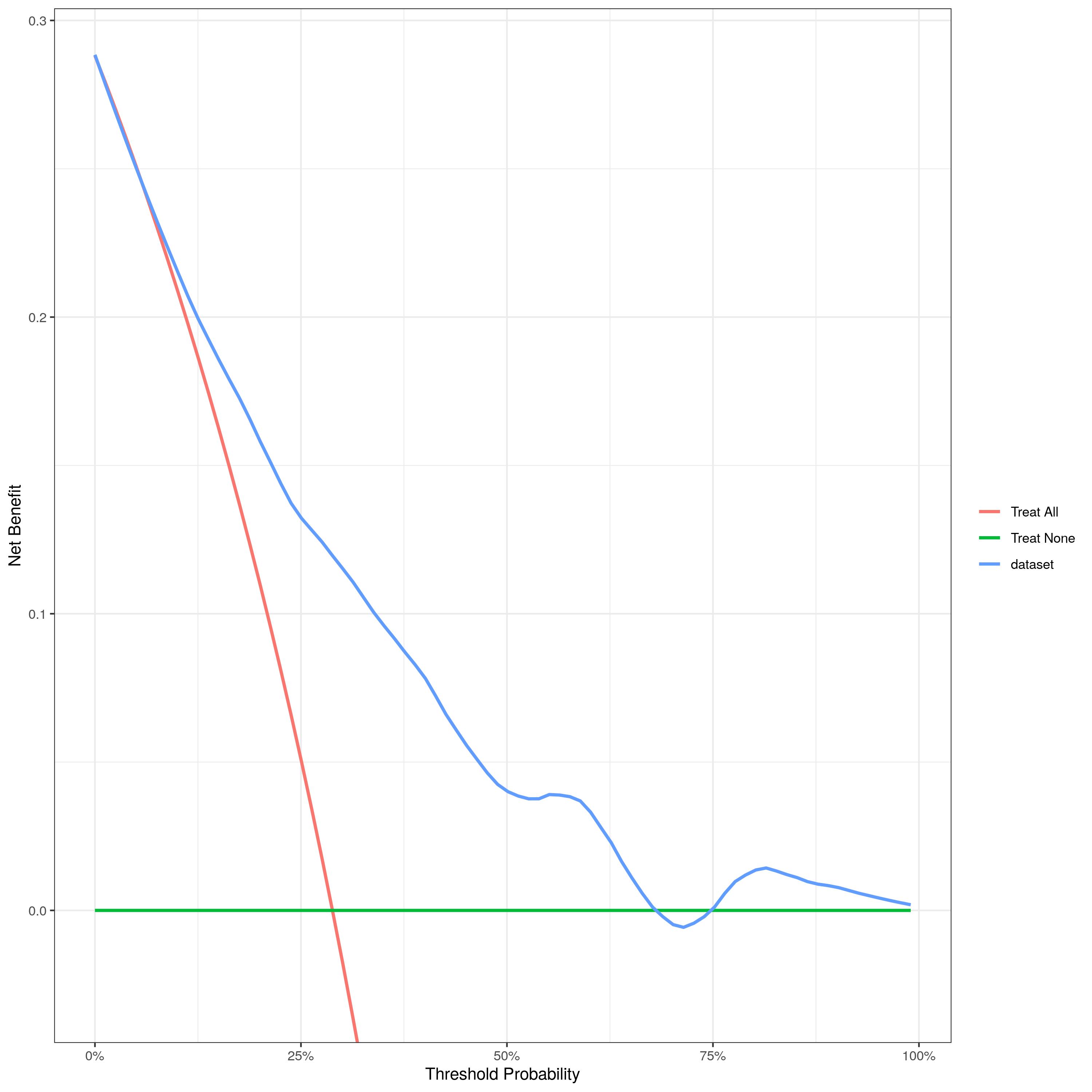

Supplement: SUPPLEMENTARY FIGURE S2 — Decision curve analysis of the predictive model for ICU mortality in patients with severe acute pancreatitis. [file Figure_2.JPEG]
